# Behavior Patterns of Antisocial Teenagers Interacting with Parents and Peers: A Longitudinal Study

**DOI:** 10.3389/fpsyg.2017.00757

**Published:** 2017-06-02

**Authors:** Francisco J. P. Cabrera, Ana del Refugio C. Herrera, San J. A. Rubalcava, Kalina I. M. Martínez

**Affiliations:** ^1^Laboratorio de Interacción Social, Departamento de Psicología, Universidad Autónoma de AguascalientesAguascalientes, Mexico; ^2^Psicología, Departamento de Ciencias Sociales, Universidad Autónoma de Ciudad JuárezCiudad Juárez, Mexico; ^3^Laboratorio de Investigación sobre Desarrollo y Contexto del Comportamiento Social, Facultad de Psicología, Universidad Nacional Autónoma de MéxicoCiudad de México, Mexico

**Keywords:** social interaction, teenagers, antisocial behavior, parenting, friendship

## Abstract

Antisocial behavior may begin during childhood and if maintained during adolescence, is likely to continue and escalate during adulthood. During adolescence, in particular, it has been established that antisocial behavior may be reinforced and shaped by exchanges between the teenager and his parents and peers, although the molecular process of these relations is as yet unknown. This paper explores the patterns of social interaction established by adolescents with and without the risk of engaging in antisocial behavior in order to understand the exchanges of them with their most important social groups, during 2 years. The study involved a sample of 70 adolescents classified into these two groups (with risk of antisocial behavior and control group). They were video-recorded interacting with one of their parents and one of their peers, independently. The interaction was done about the negotiation of conflictive conversational topics. Those video-records were registered by pairs of trained observers, using an observational catalog with nineteen behavioral categories, to know about the molecular interactional patterns characteristics. Thirty participants were evaluated only once, 30 were evaluated two times, and the other 10 were evaluated three times, the evaluations were performed annually. It was found that a higher occurrence of eye contact and use of open questions and elaborate answers appears to act as a protective factor for engaging in antisocial behavior.

## Introduction

Antisocial behavior has been defined as kind of behavior that is directed against other people, their property or breaks social rules (Garaigordobil and Maganto, 2016; Jalling et al., 2016; Garaigordobil, 2017). This type of behavior takes various forms (with different seriousness) such as lying, risky sexual practices, rule-breaking, illegal substance use and disruptive behavior such as theft, destruction, fraud, engaging in aggression (either physical or verbal), and vandalism (Patterson, 1982; Kazdin, 1987; Arce et al., 2011; Torry and Billick, 2011; Pears et al., 2016).

This range of behavior makes it a problem whose severity and frequency are a matter of concern (Pedroza, 2006, unpublished). It is usually maintained during adolescence and adulthood in individuals who displayed behavioral problems in childhood (Robins, 1986; Barkley et al., 1990; Campbell, 1991; Gaeta and Galvanovskis, 2011; Snyder et al., 2012; Alink and Egeland, 2013; Rhee et al., 2013; Çelik et al., 2016). The influence of a number of risk factors linked to the development of this type of behavior has been found, including family environment and involvement with antisocial peers (Alcázar and Bouso, 2008; Antolín et al., 2009).

As for the family environment, risk factors associated with the development of antisocial behavior include marital conflict, family stress, parental authoritarianism, parental criminality, domestic violence, social marginalization and coercive social interaction between parents and children (Loeber, 1990; Frías-Armenta et al., 2003; Quiroz et al., 2007; Antolín et al., 2009; López and Rodríguez-Arias, 2012; Rhee et al., 2013; Çelik et al., 2016).

The term “coercive” refers to the use of aversive utterances by a member of the dyad regarding the behavior of a third party, intended to modify the behavior of the latter (Patterson et al., 1989, 1992; Santoyo and López, 1990). Coercion is the result of escape contingencies and positive reinforcement of aversive events in interactions between parents and their offspring (Smith et al., 2014).

This become clear in Braga et al. (2017) meta-analysis, that included 33 studies centered in antisocial behavior in youth, authors this kind of behaviors associated with suffering aggressiveness in home; they also found that withdrawal/neglect propitiate antisocial enrolment from the adolescent.

These results coincide with Slattery and Meyers’s (2014) work; they evaluated parental monitoring, deviant peer’s association, aggression in the social environment, and behavioral problems in 503 adolescents. They found that antisocial behavior is correlated positively with the association with antisocial peers. While parental monitoring is negatively associated with behavioral problems in general, and mediates the influence of the aggression in the social environment.

In Centro-America, this kind of result has been also found in a 1599 youth sample using self-reports. It was found that the parental monitoring, conflictive family interactions, and low intimacy, were related with alcohol and drugs consumption (Obando et al., 2014).

Smith et al. (2014) got similar findings in children. They conduct a tree years’ study whit 731 dyad parent-toddler observing teaching task, playing, and preparing/eating time, finding a strong relationship between coercive interactions among adult and child and the escalation and generalization of the aggressive behavior in the toddler.

Other work has also found that low quality in family relationships, lack of emotional expression (in parents) and intrusive parental practices can lead social isolation during adolescence (Rovis et al., 2015), this isolation, by itself implies an important risk to be a bullying victim (Aguilera et al., 2013), and rule breaking, vandalism and alcohol consumption (Ettekal and Ladd, 2015).

This may be due to the fact that parents of antisocial teenagers and children often lack the necessary skills to respond appropriately to their children’s behavior, using aggressive techniques to modify undesirable behavior, modeling and reinforcing these behaviors over prosocial ones (Torry and Billick, 2011), and thereby establishing patterns of coercive interaction that are replicated in other settings (Patterson et al., 1992; Quiroz et al., 2007; Bowker et al., 2016).

On the other hand, it had been found that positive affect, parental monitoring and responsiveness will facilitate the linking with prosocial peers (Bowker et al., 2016), while adolescents whose parents use harsh discipline tend to link with antisocial peers, as Li et al. (2015) found in their study, in which participated 993 same sex twin’s pairs (13.72-year-old) and their parents. In this work the researcher evaluated peer filiation, discipline and parent’s negative emotional expression through self-report (questionnaires) from both parents and adolescents. They found that adolescents, whose parents use harsh discipline, tend to link with antisocial peers. This became important taking in account that another important risk factor, especially during adolescence, is the teenagers’ interaction with peers, particularly when the latter are antisocial (Snyder et al., 2012), since antisocial behavior in these groups is modeled, and positively reinforced (Ayala et al., 2002; Snyder and Stoolmiller, 2002).

In terms of the peers’ influence, a 9 years’ longitudinal work, with 998 adolescents has found an increase in deviant behavior with the social reinforcement (of this kind of behavior) provided by peers (Dishion et al., 2012), they also fund that isolated individuals will tend to connect with antisocial peers during adolescence.

Ettekal and Ladd (2015) got similar outcomes in a longitudinal study whit 383 children followed 5 to 14 years old (in nine waves), parents, professor and the children respond questioners about rule breaking, disruptive/aggressive behavior and peer deviant friendship, respectively, while classmates provide information about peer rejection. This work shows that disruptive/aggressive behavior during childhood is a risk for prosocial peer rejecting and later for deviant peers linking and early adolescence rule breaking behavior and aggressive behavior. Consistent with this work, Carlo et al. (2014), using a questionnaire with 666 adolescents, found that prosocial peer filiation were negatively related to antisocial social behavior; Van Ryzin and Dishion (2014), make a 7 years’ longitudinal study, found a strong relationship between drug use and affiliation with deviant peers.

Although these factors have been identified, most of the work has been conducted using questionnaires so the molecular interaction process that encourages the presence and maintenance of antisocial behavior remain unknown (Reid et al., 2002). A molecular approach would allow to assess the triple relationship of contingency in terms of antecedents, behavior and consequences that strengthen or weaken the conduct, as molecular studies are characterized by a detailed comportment analysis, behavior by behavior, moment to moment, while molar analysis show us general aspects of behavior (Anguera, 2003; Anguera et al., 2007; Pellón, 2013).

Given the importance of these two major spheres of interaction, it is essential to understand the micro process that takes place during teenagers’ interaction with parents and peers, and to determine whether there are differences in interaction patterns at different stages of development. Taking in account the findings in the literature, the hypothesis of this work is that there will be differences between the social interactions held in parent-teen in risk of antisocial behavior and parent-teen without risk. It is also expected that those differences will found in adolescent-peer interactions; the adolescents in risk would have more conflictive and less responsive social exchanges in both cases.

One method that makes it possible to identify these processes is the direct observation of behavior, since it makes it possible to pinpoint both the coercive process and the development of the trajectories of antisocial behavior (Anguera et al., 2007). This study therefore focused on understanding the interaction patterns of adolescents reported as engaging in any form of antisocial behavior by their teachers as well as possible differences from teenagers matched for age, sex and school year, through the implementation of an interactive task, in a longitudinal study.

## Materials and Methods

### Participants

A total of 70 high school students participated voluntarily, in the period from 2011 to 2013. Participants had a mean of 13 years old (*SD* = 8 months) at the start of the study, and 24 students was men and the rest were women. Of the total sample, 35 students were reported by their teachers as being at risk for antisocial behavior (risk group, RG) of which 15 participated in a single evaluation, 15 in two evaluations and the remainder in three evaluations. The evaluations were performed annually.

The other 35 evaluated students were chosen as controls using two criteria: (1) were matched with participants in at risk-group by age, sex and school year, and (2) they had to be students who were not reported by teachers in the behavioral risk categories. The edge and gender was the same in risk and control groups. Because the study suffered experimental death, different numbers of participants in RG were evaluated during each wave of evaluation, then, each subject in the control group was evaluated so many times as was the person in the RG to which was matched.

Additionally, in this study participated, in each evaluation period, one of the teenagers’ parents and one of their friends, which participated voluntarily. The parents were constant if the evaluation occurred two or three times, but in some cases the teenager select a different friend in the next evaluation of teenager-peer interaction.

### Scenario

In order to obtain videos on dyadic social interaction, three different scenarios were used: (1) The Psychology Care and Research Unit at the Autonomous University of Aguascalientes, in a 3 × 4 meter cubicle with a table and two armchairs; (2) an area in high school facilitated by authorities, the scholar auditorium; and (3) the teenager’s parents’ house, where the activities were done in a room in which was collocated a table and chairs. In each setting, we ensured adequate lighting and privacy.

### Materials and Instruments

- Informed consent. Printed document explaining the purpose of the research, as well as the activities carried out, and requesting parent’s authorization for the inclusion of their teenager son/daughter to participate in the study. This document lets the parents know about the possibility that they need to participate either.

- Observer XT^®^ version 9. Computer program designed to record observational data and permits the use of behavioral categories to code behavioral variation in the course of social interaction.

- List of topics. List of 37 conversation topics and a final section in which other topics may be added and classified as conflict or no-conflict, made *ex profeso* for this investigation. The topics were relation with family members, relation with parents, home rules, time of arrival home, permissions, friends, best friend(s), boyfriend/girlfriend, school teachers, school mates, school grades, subjects failed, homework, future studies, eating habits, study habits, reading habits, sporting activities, personal belongings cleaning, room cleaning, housework, dressing way, way of talking, weekends, movie theater, party with friends, television programs, outings with the family, music, vacations, drugs, alcohol, money, work, sexuality, religion, politics.

- Behavioral Catalog for Teachers. Check list of 21 behaviors, based on diagnostic criteria for antisocial behavior from the fourth edition of the Diagnostic and Statistical Manual of Mental Disorders in its fourth edition (American Psychiatric Association [APA], 2002).

- Catalog of Direct Observation of Negotiation (Pedroza et al., 2012). Comprising 19 categories providing a detailed account of eye contact and the content of the utterances made by each member of the dyad during the negotiation process (for access to full catalog, see reference). The categories were evaluated by experts in the area of social exchange and some of them were re-defined after its use in the classification of social exchanges occurred in son of the videos. Experts approve the final version with 100% of concordance.

- Theme 5^®^.- Computer program designed to locate behavioral patterns hidden from the naked eye; fed with continuous observational data.

- SPSS 20. -Software that makes it possible to process statistical data.

### Procedure

The researchers contacted the directors of a public middle school, requesting their authorization to undertake the research activities. Teachers were informed of the project, after which the Behavioral Catalog for Teachers was applied. Once the data had been obtained, students who were eligible for participation were identified. These students and their parents were contacted through the institution, the objectives of the research were explained, and they were asked to sign the informed consent form.

The evaluations spend around 30 min, in which each dyad (teenager-parent or teenager-peer) have two activities, first they were asked to make the classification of the issues of the List of topics as conflictive or no conflictive item of talking. In the second activity, the dyad was asked to negotiate about of the most conflictive issues previously classified, for 20-min. The evaluations were performed two more times, annually.

These interactions were video recorded, and after that were behavioral-categorized using the Catalog of Direct Observation of Negotiation, through XT^®^ OBSERVER the observations were done for two observers previously training for such labor, and that obtained more than 0.70 of concordance inter-observers three consecutive times, evaluated with the statistic Kappa de Cohen. Finally, the obtained behavioral sequence was used to search for hidden patterns through THEME 5^®^. A statistical analysis of the data was undertaken through SPSS 20 version, considering the teenager’s experimental group, school year and time of assessment, and noting the rate of displays of aggressive behavior and average duration.

## Results

In order to analyze the data obtained in the first assessment, we analyzed the behavior displayed by the teenagers in their interaction with their peers, considering whether they belonged to the RG or the matched-control group (CG) and the students’ school year, using the Kruskal–Wallis Test. Through these analyses, significant differences were found in *agreement* behavior, which, in the case of the teenagers, was displayed by the control group in the first grade, with an average rate of 0.03 (*SD* = 0.01). Regarding interaction with peers, no significant differences were observed in the population analyzed for state behaviors.

Regarding the rest of the behaviors analyzed in the teenagers’ interaction with their parents (*p* < 0.05), a number of differences were observed when they engaged in *debating* behavior (verbalizations that explain or justify facts). A multiple analysis showed that the differences occurred between teenagers in the first and second grade, regardless of the experimental group to which they belonged, with those in the second grade displaying this behavior to the greatest extent, with $\overset{¯}{X}$ = 0.72 (*SD* = 0.1) in RG, and $\overset{¯}{X}$ = 0.65 (*SD* = 0.12) in CG, whereas in the first grade, the results were as follows: $\overset{¯}{X}$ = 0.29 (*SD* = 0.12) in RG, and $\overset{¯}{X}$ = 0.13 (*SD* = 0.21).

In the case of the students’ parents, significant differences were found regarding *negative verbal behavior, containment*, and *hostile containment.* Multivariate analysis revealed that these differences were due to the utterance rate of parents of RG teenagers in second grade. In particular, this group of parents engaged in these three behaviors more frequently than parents of those in the control group, in second grade. In the case of containment behavior, differences were also observed with regard to the parents of teenagers in the control group in the first grade of middle school, with *p < = 0.05* in all cases.

During the second evaluation, Kruskal–Wallis analysis showed that the utterance rate of RG and CG students in interaction with their peers differed significantly as regards *negative verbal behavior about third parties* and *termination*, which involves verbalizing disapproval of personal or situational behaviors of various individuals outside the dyad in interaction, and a specific request to change topic from the conflictive issue being discussed. Multivariate analysis showed that differences were observed between the data on the teenagers in RG in second grade and those in CG in the second and third grades, the latter, who expressed greater disapproval of third parties with an average rate of 0.37 (*SD* = 0.45) and 0.30 (*SD* = 0.28) utterances, respectively, compared with an average of 0.09 (*SD* = 0.07) utterances by adolescents in the at-RG in second grade. As regards *termination* behavior, a difference was only observed between students in the at-RG in third grade and students in the control group in the second grade of middle school, with the latter displaying the highest rate of requests to change topic. Data on the mean and standard deviation of both behavioral categories are given in **Table 1**.

**Table 1:** Descriptive statistics of negative verbal and termination behaviors engaged in by teenagers in interaction with peers.

Behavior	Experimental group	School year	Mean	Standard deviation	*N*
NEGATIVE VERBAL COMMENTS ABOUT THIRD PARTIES	At-risk group	First grade	0.00	0.00	2
		Second grade	0.09	0.07	9
		Third grade	0.13	0.10	10
	Control group	First grade	0.37	0.45	4
		Second grade	0.30	0.28	14

TERMINATION	At-risk group	First grade	0.81	0.71	2
		Second grade	0.40	0.57	9
		Third grade	0.26	0.26	10
	Control group	First grade	0.64	0.15	4
		Second grade	0.62	0.36	14
		Third grade	0.28	0.23	4


*The mean reported is about the rate of emission by minute.*

A comparison of the data on the behaviors displayed by teenagers during the interaction with their parents, considering the school year, yielded significant differences in *clarification* behavior, engaged in at a higher rate of occurrence per minute by teenagers in the control group in second grade (=1, DE = 0.68) compared to those in first grade RG ($\overset{¯}{X}$ = 0.31, *SD* = 0.21); second grade RG ($\overset{¯}{X}$ = 0.28, *SD* = 0.15); third grade RG ($\overset{¯}{X}$ = 0.39, *SD* = 0.27); first grade CG ($\overset{¯}{X}$ = 0.71, *SD* = 0.43) and the third grade group CG ($\overset{¯}{X}$ = 0.31, *SD* = 0.28).

Regarding the time spent by teenagers on issues outside the experimental task, significant differences were found when the experimental group was considered. RG spent more time on this category with an average time per minute of 0.05 (*SD* = 0.07) as opposed to the mean of 0.008 (*SD* = 0.03) by the control group. It was also found that teenagers in the control group spent more time per minute in *eye contact* ($\overset{¯}{X}$ = 0.66, *SD* = 0.31) than those in the at-RG ($\overset{¯}{X}$ = 0.41, *SD* = 0.28), with an alpha < 0.01.

As for the parents, the second evaluation revealed differences in the number of utterances per minute involving *hostile containment*. The application of multivariate analysis showed that the differences were due to the scores of parents of RG teenagers in third grade when compared with parents of GI adolescents in second grade and those of teenagers in the control group in the second grade. This last group displayed the lowest emission rate per minute with 0.01 and 0.03, respectively.

The time spent on verbal behavior also varied among the parents of the adolescents described in the preceding paragraph, particularly as regards *conversation topic* and *being silent*. Differences were due to the average duration of the utterances of parents of RG teenagers in second grade, who verbalized the topics in the task a third of the time, whereas the other participants showed the reverse pattern. By comparing the data that only considered the experimental group, it was found that the parents of RG teens spent less time addressing conflictive issues ($\overset{¯}{X}$ = 11 min) than the parents of CG teens ($\overset{¯}{X}$ = 16 min), *p* < 0.005, but spent longer in silence, *p* < 0.01 (RG $\overset{¯}{X}$ = 8.5 min; and CG $\overset{¯}{X}$ = 4 min).

With regard to the third application, Kruskal–Wallis’s non-parametrical statistical test was used to determine whether students in the two experimental groups in second and third grade showed significant differences. No significant differences were found in adolescents’ behavior in their interaction with peers. It is worth noting that behaviors involving a positive assessment of the other person with whom one is interacting *(positive verbal)* were only observed in participants in the control group; the opposite happened when people absent at the time of the negotiation were evaluated. On the other hand, behaviors that involve offering to make changes (*concession*), giving in to the demands of the other person (*hostile concession*) and requesting a change in the other person coercively (*hostile containment*) were not observed in teenagers during their interaction with their peers. Moreover, although the differences were not significant, a trend of increased time spent on addressing the issues in the experimental task and eye contact was detected in teenagers in the control group, who used it just over half the time in the former behavior and about 80% in the latter, compared with 30 and 50% in the at-RG for *conversation topic and eye contact*, respectively.

As for interaction with parents, teenagers in the control group were the only ones who engaged in *positive verbal behavior* with an average of approximately one utterance per video in the case of participants in second grade and two utterances per video in those in third grade. *Positive verbalization about third parties* occurred with the same average although in this case, it was engaged in by second graders in the at-RG and by third graders in the control group. *Hostile concession* and *agreement behaviors* were not displayed by second graders in either experimental group. The Kruskal–Wallis statistical test only revealed significant differences in the *termination* behavior, with the at-RG displaying this behavior to a greater extent. A multivariate analysis revealed differences between students in the at-RG in third grade and students in the same experimental group but in second grade of middle school and those in the control group in second grade, with a probability of error of less than 0.05.

Although no significant differences emerged regarding state behaviors, it was found that participants spent more time without making verbal utterances, approximately 80% of the time available for interaction, though eye contact is more common in adolescents in the control group and present in over 60% of the time of interaction as opposed to 35% in the at-RG.

Regarding parents, only parents of third grade adolescents in the at-risk third group engaged in *hostile concession*, with an average utterance rate of 0.02 (*SD* = 0.03). Conversely, *hostile containment behavior* was only displayed by parents of GI adolescents in the second grade ($\overset{¯}{X}$ = 0.15, *SD* = 0.10) and CG third grade teenagers ($\overset{¯}{X}$ = 0.03, *SD* = 0.04). *Agreement* behavior was not displayed by these participants.

Although no significant differences occurred in *questioning, clarifying and simple answer* behaviors, involving requests for information, verbalizations that provide extensive information at the request of the other person and brief verbalizations (even monosyllables) given in response to the request for information, it was observed that these behaviors display the highest rate of utterances per minute, as shown in **Table 2**.

**Table 2:** Utterance rate per minute of ask, clarify, and simple answer behaviors.

	Ask		Clarify		Simple answer	
			
Participant	$\overset{¯}{X}$	*SD*	$\overset{¯}{X}$	*SD*	$\overset{¯}{X}$	*SD*
Teenager in interaction with parent	0.45	0.45	0.55	0.51	0.90	0.75
Father	1.43	1.00	0.34	0.29	0.46	0.34
Teenager in interaction with peer	1.97	1.32	0.16	0.28	0.13	0.16
Peer	1.06	0.67	0.47	0.47	0.66	0.56


Lastly, hidden interactions patterns were sought throughout the evaluations. This analysis yielded an average of 20 patterns per group of observations submitted. This paper therefore only describes the most significant patterns found for each analysis undertaken. It should be noted that all the results obtained through this method have a *p <* = 0.0001.

**FIGURE 1:**
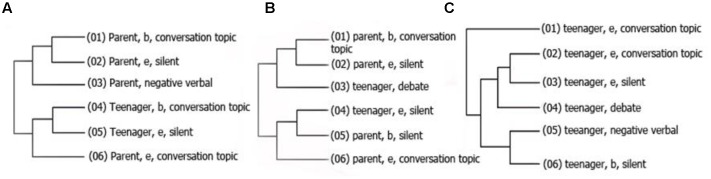
**Graphic representation of three hidden patterns** **(A–C)** of interaction occurred between RG teenagers and their parents. Patterns shows the sequence of presentation of the behaviors which were found associated significantly, moreover, the lines connect the conducts that occur more frequency relates with each other. Numbers in parenthesis indicate the sequence of the patter and the letters *b* and *e* were used to indicate if the behavior presented after was beginning (b) or ending (e), and appear only if the behavior was a state behavior.

Regarding all the observations and the participants separated only by experimental group (at-risk/matched), the following behavioral patterns were identified in the interactions between adolescents at risk for antisocial behavior and their parents: the first pattern (**Figure 1A**) shows that there is a high probability that once a parent belonging to group 1 begins talking about the topic of discussion (conversation topic), s/he will also utter expressions of disapproval about the teenager’s behavior (negative verbal behavior); subsequently, the teenager will begin discussing the topic in hand.

The second (**Figure 1B**) and the third (**Figure 1C**) relate to the teenager’s behavior when s/he begins discussing the subject. There is a high probability that once the teenager begins to address the conflictive issue, s/he will begin to engage in *debating* behavior (which involves justifying acts or behavior), whereby s/he ends his or her verbal behavior and the parent takes up the conversation.

**FIGURE 2:**
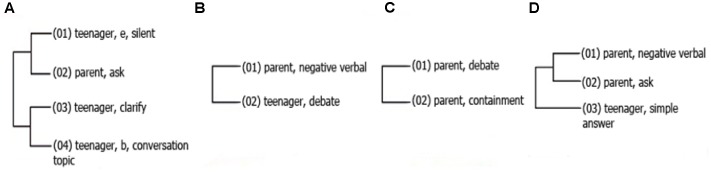
**Graphic representation of hidden interaction patterns in exchanges between CG teenagers and parents.** This figure presents four patterns called **(A–D)**. Patterns shows the sequence of presentation of the behaviors which were found associated significantly, moreover, the lines connect the conducts that occur more frequency relates with each other. Numbers in parenthesis indicate the sequence of the pattern and the letters *b* and *e* were used to indicate if the behavior presented after was beginning (b) or ending (e), and appear only if the behavior was a state behavior.

Three significant patterns emerged in the comments made by adolescents engaged in interaction with their parents. The first (**Figure 2A**) shows that when the teenager is silent, the father will seek information (*ask*), which triggers an elaborate response made by the teenager (*clarify*), which leads to a resumption of the conversation.

The second pattern (**Figure 2B**) shows the link between the expressions of disapproval by the parent and the justifications by the teenager. Another important pattern found (**Figure 2C**) shows the parent’s use of expressions designed to justify his or her own behavior prior to the request for a change in the teenager’s behavior.

**FIGURE 3:**
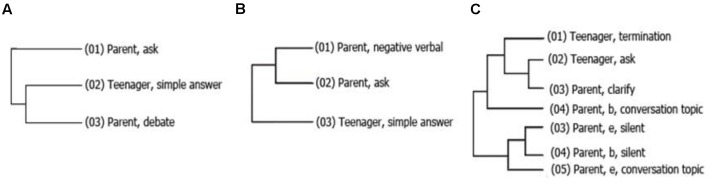
**Hidden patterns found in CG** **(A,C)** and RG **(B)** interactions with parents related to the way they ask and give for information. The patterns of adolescents of control group interacting with parents shows a clear interchange of bidirectional information, that is, they used consistently the behavior of ask, while the risk group shows that the parents use aversive events (negative verbal) before asking for information, and the behavior of ask for information didn’t appear as part of the teenager’s patterns.

Regarding the patterns found in the different cohorts, in the interactions between first grade adolescents and parents in the at-RG during the first evaluation, a pattern emerged of expressions of disapproval (negative verbal behavior) by the parent, followed by a search for information, which only elicited a short response from the teenager (**Figure 2D**). Conversely, in the control group, patterns were found that included a search for information by the parent (**Figure 3A**) which elicited a short response from the teenager, followed by the use of expressions designed to present arguments related to the topic in hand.

As for the dyads in the control group in second grade in the first evaluation, it was found that the parents expressed disapproval, they continued searching for information to which the teenager gave short replies (**Figure 3B**). It was also found that after s/he began discussing the topic, the teenager uttered expressions designed to explain her or his behavior (**Figure 3C**).

**FIGURE 4:**
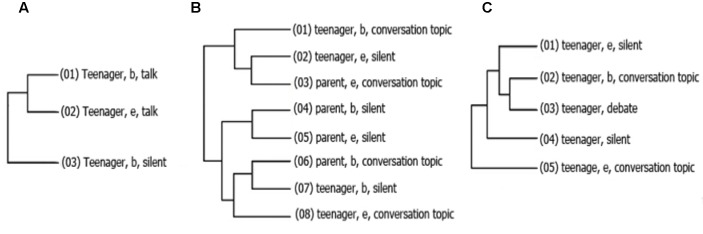
**Hidden patterns found in exchanges between RG teenagers and parents.** Even though the patterns were found in interactional situations, the patterns **(A,C)** shows that teenagers behavior is consistent itself, regardless the parents behavior. The pattern **(B)** shows that the talking of one of the dyad members was associated with the end of talking of the other.

During the 2nd year of assessment, participants from the first, second and third grade were included. In the interactions between second grade adolescents and their peers in the control group, a pattern emerged showing the path of the conversation (**Figure 4A**). In the at-RG, this group contains fewer elements (**Figure 4B**) and only involved one of the participants (this pattern was found in both the adolescent and the peer).

During the 3rd year of evaluation, second and third grade students participated. An important pattern found among second-grade participants in the at-RG interacting with their parents included silence by both the parent and the teenager after the topic had been mentioned by the other person (**Figure 4C**). Another pattern involved the process of negotiation, whereby the teenager *debates*, after beginning to discuss a particular conversation topic.

At the same time, second-grade participants in the control group interacting with their parents showed a pattern whereby the parent interrupted eye contact only after it had been interrupted by the teenager. Another pattern shows that the utterance of expressions of disapproval by the parent (negative verbal) occurs after the parent changes the topic of conversation (termination).

Lastly, among the participants belonging to the at-RG who were in third grade during the third wave of evaluation, a total of 16 interaction patterns were found. Of these, the most important shows that in response to negative verbal behavior by the parent, the teenager begins to engage in debate. Another pattern shows that participants ask questions and provide clarification immediately afterward. It is worth noting that both patterns occur in both directions.

In relation to the patterns of interaction found using Theme in the interactions of adolescents with their peers, 72 patterns were found, of which the following are the most complex for each group.

**FIGURE 5:**
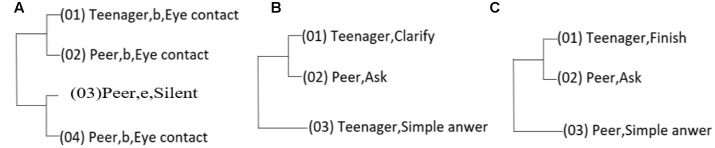
**Hidden patterns found in RG adolescent-peer interactions using the software THEME.** Numbers in parenthesis indicate the sequence of the pattern and the letters *b* and *e* were used to indicate if the behavior presented after was beginning (b) or ending (e), and appear only if the behavior was a state behavior. The **(A–C)** were used to indicate each one of the patterns.

A pattern of **Figure 5A** shows the changes in Eye contact; where the adolescent begins *eye contact*, that was follow by the peer beginning eye contact, later. **Figure 5B** shows the use of *clarify* by the teenager, follow by a question form the peer that lead to a *simple answer* by the teenager. The **Figure 5C** displays a pattern that has a topic transition (*finish*) made by de teenager, that lead a question by peer; that question (*ask*) is responded by a *simple answer*.

**FIGURE 6:**
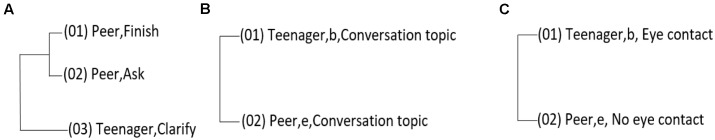
**Interactional hidden patterns found between CG adolescents and their peers.** The first pattern **(A)** shows a sequence of inquiry; the second pattern **(B)** shows adolescent and peer within the topic of conversation, where the teenager talks and the other opts to keep silence; and the third pattern **(C)** reflex the difficul to mantence mutual eyes contact.

In the adolescents CG interacting with peers (**Figure 6**) a first pattern (**Figure 6A**), shows, the changes in the central topic of discussion, where change were made by de peer (finish), using a question as topic changer (ask). The **Figure 6B** contains, a pattern of the using of word, that shows that once de teenager begins (b) to talk about a conversation topic, the peer would start silence (e [end] Conversation topic). Meanwhile, **Figure 6C**, shows that the beginning of *eye contact* from the teenager, leads to *eye contact* by the peer.

## Discussion

The mean objective of the present study was to analyze social interaction patterns of teenagers at a micro level, to try of explain how differ the behaviors that occur in teenagers with risk of antisocial behavior versus teenagers without such risk, when they interact with their parents and friends, and how this difference affect the maintenance of teenager’s behavior.

The differences between adolescents who are at-risk or not at-risk of antisocial behavior in the interactions with their parents and their peers are reflected in the time each of the groups spends discussing relevant issues (conversational topics), with control group participants spending longer on this behavior. The same occurs with eye contact. The higher rates of occurrence of these behaviors indicate greater exchange in the dyads in the control group.

With regard to interactions between adolescents in both groups and their peers, the fact that no significant differences have been found in the utterance rates may indicate that the greatest difference lies in the type of activities in which they jointly engage (prosocial or antisocial), which are therefore reinforced and modeled (Reid et al., 2002; Snyder and Stoolmiller, 2002). Moreover, the hidden patterns identified in teenager-peer interaction show a little complexity, that is, they talked about the conversational topics using answers and given answers, but didn’t used complex behaviors as a constant, such as *debating* or *negative verbal* or categories related to negotiation processes (*concession*, *containment*, or *agreement*).

Meanwhile, the micro-analysis of data in terms of the search for hidden patterns showed that the interaction that occurred between adolescents and their peers and parents is qualitatively different, according to previous studies (Ayala et al., 2002; Ettekal and Ladd, 2015). In this regard, observations during the 1st year of evaluation showed that regardless of their school year or experimental group, interaction between adolescents and their peers shows a pattern in which addressing issues is associated with the request for information and subsequently obtaining this information, followed in the case of at-risk students, by a change of subject. This pattern may imply an exchange limited to obtaining data without questioning them, meaning that the exchange of views is not reciprocal. In other words, in addressing various issues, each student questions the other about a different issue, meaning that it is not necessary to share views on a topic when they diverge. If this strategy avoids conflict, it decreases the likelihood of the breakdown of a relationship, which is an indicator of coercion (Reid et al., 2002; Van Ryzin and Dishion, 2014).

These results about teenager-peers interaction give partial confirmation to our hypothesis. On the one hand, more time is spends discussing relevant issues and having visual contact by CG, but, on the other hand, the king of contend of such discussion did not differ significantly from RG. We had argued that the difference could lies in the type of activities they engage together. However, more interaction time of teenagers in CG with their peers let a mayor possibility of practice another behavior that could be efficient in negotiation situations. Future research on older adolescents is necessary to corroborate whether negotiation behaviors improve.

An analysis of the period when the adolescents were evaluated revealed changes in the rates of occurrence of a particular behavior, particularly at-risk family conditions, reflected in the rates of *negative verbal, containment and hostile containment* behavior in parents of RG teenagers in second grade during the 1st year of assessment, when students had been exposed to anti-social peers for longer (Frías-Armenta et al., 2003; Antolín et al., 2009). These rates were higher than those displayed by parents of CG students in first and second grade, which may suggest that parents of matched adolescents use less rigid discipline.

In the case of the at-RG of both first and second grade students in social interaction with parents, addressing conflictive issues follows a behavioral pattern in which parents present an aversive stimulus (*negative verbal*) that is associated with a subsequent request for information, which makes it possible to obtain *short answers* from the teenager, whereas in the case of the control group, the request for information by the parent is not associated with the prior presentation of an aversive stimulus and elicits elaborate answers from the teenager. Although the control group shows a behavioral pattern in which *asking* on the part of the parent is associated with a *simple answer* from the teenager, in this pattern, the use of a *simple answer* is associated with subsequent engagement in *debating behavior*. These differences in the exchange structure may indicate the use of escape contingencies by the at-RG of teenagers (Reid et al., 2002; Snyder and Stoolmiller, 2002; Smith et al., 2014), while in the control group it represents the continuity of discussion, that had more posibiliti of *agreement*, as data indicated.

During the 2nd year of evaluation, a striking feature is the use of the disapproval of third parties’ behavior by RG teenagers in the third grade when interacting with their peers, which may suggest that the issue of aggression toward others plays an important role in RG teenagers’ exchanges with their peers and, therefore their affiliation with antisocial peers, which constitutes a risk factor for engaging in antisocial behavior (Snyder et al., 2012).

As for teenager-parent interactions in the second evaluation, it is important to highlight the use of elaborate responses (*clarify*) by the parents of CG adolescents in second grade, which was significantly higher than the rate of RG participants in all grades who participated in this evaluation. This situation may indicate the use of good parenting skills expressed through the use of successful information seeking strategies by parents and low rates of aggressive requests (*hostil*e *containment*) and the availability to interact and low use of avoidance strategies by adolescents, which may explain the absence of antisocial behavior in this group (Patterson et al., 1992; Torry and Billick, 2011).

Regarding the 3rd year of evaluation, in teenagers’ interaction with their parents, it is striking that CG teenagers express approval of their parents’ behavior (*positive verbal*); this may indicate the good state of interactions between adolescents and parents in this group and therefore the absence of coercive interactions between them. Conversely, the constant change of topic of conversation (*termination*) by RG members may indicate escape strategies, as well as a low rate of exchange. These data, coupled with parents’ requests to change the topic, contingent on the use of negative consequences (*hostile containment*) indicate the presence of a coercive process between the parents and adolescents in this group, which implies risk factors for adolescents to engage in antisocial behavior (Patterson et al., 1989, 1992; Santoyo and López, 1990). Parents in CG not only had a prosocial interaction with their teenage children but also modeled to them how act when a conflict issue is addressed.

By undertaking the analysis considering data from all the evaluations and only differentiating participants by experimental group, it was found that in GI parent-child exchanges, beginning to address a specific topic was associated with the utterance of *negative verbal* phrases by both by parent and teenager, which implies the constant disparagement of the person with whom one is interacting. In the case of adolescents, this disparagement is associated with the previous occurrence of verbalization in which the adolescent justifies his or her behavior (*debate*). This situation shows the use of aversive utterances in the exchanges, which may serve as a predictor of antisocial behavior since the display of physical and verbal aggression during childhood and adolescence has been associated with the development of this type of problems in the literature (Gaeta and Galvanovskis, 2011; Snyder et al., 2012; Alink and Egeland, 2013; Rhee et al., 2013; Garaigordobil and Maganto, 2016; Jalling et al., 2016).

These data also show that the behavior modeled by RG parents is aggressive, since the display of verbal disapproval associated with the start of verbal behavior oriented toward the chosen conversation topic (*conversation topic*) by the father is also a risk factor for the development of antisocial behavior as is parents’ authoritarianism (Loeber, 1990; Frías-Armenta et al., 2003; Quiroz et al., 2007; Antolín et al., 2009; López and Rodríguez-Arias, 2012).

The fact that RG teenagers’ acts are not associated with the expression of disapproval by the parent could also denote the use of avoidance strategies by the teenager and thus the development of coercive interactions (Patterson et al., 1989, 1992; Santoyo and López, 1990).

The use of verbalizations in which the disapproval of adolescent behavior (*negative verbal*), prescribing changes in the adolescent’s behavior (*containment*) and the anticipation of negative consequences if the prescribed changes are not made (*hostile containment*) create an inauspicious setting for negotiation, which explains the absence of engagement in “agreement” by parents and their offspring in the at-RG.

A different situation was identified in the case of CG. Parents’ engagement in *negative verbal* behavior is associated with the justification of facts (*debate*) by adolescents. Moreover, the utterance of parent’s disapproval was associated with previous use of *debating* behavior which may indicate the use of inductive behavior by parents, and the prescription of previously justified changes.

Another difference found in the parent-child interaction patterns between the at-risk and control groups is that in the latter, the questions used by parents are not only associated with obtaining elaborate answers from the teenager (*clarification*), but also result in the continuation of the conversation, allowing greater exchange between members of the dyad, which could be acting as protection factor (Quiroz et al., 2007; Bowker et al., 2016).

The more complexity patterns of teenager-parent interaction in CG were principally related to parents’ behavior. Previous research had found that parental monitoring and responsiveness was associated with prosocial behaviors of the children (Obando et al., 2014; Slattery and Meyers, 2014; Bowker et al., 2016), in this investigation we had found that the principal aspect of parent-teenager exchanges was related to the presentation of a more varied behavioral repertoire by parents, corroborating our hypothesis about it.

It is important to note that this paper sheds light on the molecular process (Reid et al., 2002) which contributes to the emergence and maintenance of antisocial behavior in adolescents, through the identification of specific behavior patterns in adolescents at risk antisocial behavior in their interaction with their parents and peers, which differ from the rates and behavioral patterns engaged in by teenage-parent dyads which are not at risk of antisocial behavior. Since this is a cohort study, it is important to stress the importance of longitudinal studies covering this developmental stage (adolescence).

## Ethics Statement

This study was carried out in accordance with the recommendations of “Mexican Society of Psychology, and bioethics committe of Universidad Autónoma de Aguascalientes,” with written informed consent from all subjects. All subjects gave written informed consent in accordance with the Declaration of Helsinki. The protocol was approved by “the bioethics committe of Universidad Autónoma de Aguascalientes.”

## Author Contributions

FC was the main researcher of this work; he provided de original idea and organize de research group. Later FC and AH work together on the design of the project, and with SR and KM they organized the work in field and in the training of the research assistants. FC, AH, SR, and KM had a role in the collecting data process and in the analysis and interpretation of the final data. AH and SR wrote the first draft, that late was developed by AH, FC, SR, and KM by revising and rewriting the contents. All of them worked on the final draft and agrees to be accountable for all aspects of the work in ensuring that questions related to the accuracy or integrity of any part of the work are appropriately investigated and resolved.

## Conflict of Interest Statement

The authors declare that the research was conducted in the absence of any commercial or financial relationships that could be construed as a potential conflict of interest.
